# The Relationship between Pain and Spasticity and Tell-Tale Signs of Pain in Children with Cerebral Palsy

**DOI:** 10.3390/toxins15020152

**Published:** 2023-02-13

**Authors:** Christian Wong

**Affiliations:** 1 Department of Orthopedic Surgery, University Hospital of Copenhagen, 2650 Hvidovre, Denmark; cwon0003@regionh.dk; Tel.: +45-35459411; 2 Department of Orthopedic Surgery, University Hospital of Copenhagen, Rigshospitalet, 2100 Copenhagen, Denmark

**Keywords:** pain, cerebral palsy, children, spasticity, clinical characteristics/tell-tale signs of pain, parental perception of pain, AbobotulinumtoxinA

## Abstract

Pain and quality of life are closely interrelated in children with cerebral palsy (CCP). Even though 67% of CCP experience pain, it is overlooked and untreated. In this study, our purpose was two-fold: first, to examine the relationship between pain and spasticity by evaluating the effects of AbobotulinumtoxinA/Dysport (BoNT), and second, to describe the symptoms and location of pain in CCP. The subjects were 22 CCP in at least moderate pain. They were evaluated for spasticity by the modified Ashworth and Tardieu scale and for pain by the r-FLACC and the pediatric pain profile. After one injection of BoNT, the subjects were re-evaluated. We found a significant reduction in pain, but no significant relationship between the reduction of pain and spasticity. We found no association between the dose of BoNT and pain or spasticity. Pain in the lower extremity was located primarily in the hip region. The effect of ultrasound-guided intermuscular injections of BoNT suggests that pain in CCP has an extra-articular component. We found that pain in CCP manifests as specific tell-tale signs and problems in daily living. In conclusion, we found no relationship between pain and spasticity. Signs and manifestations of pain are described in detail. Lower extremity (hip) pain seems to have a soft tissue/extra-articular component.

## 1. Introduction

Cerebral palsy (CP) is the most common inborn neurological disease, with a prevalence of 2.4 per 1000 live births [1]. One of the first apparent signs of CP in children is muscle spasticity, but other sensory deficits such as musculoskeletal and neuropathic pain should be counted among the mosaic of symptoms in children with cerebral palsy (CCP) when they are evaluated for treatment [2,3]. Caregivers report that up to 67% of CCP are in pain [4,5], thus pain is a significant clinical problem [6]. The incidence increases with age and the severity of the disease [7], and it is often presumed in both research and clinical practice that there is a relationship between spasticity/muscle contractures/joint deformity and pain in both children and adults [8,9,10,11,12,13,14,15,16,17,18,19]. However, the interrelationship is not fully understood, and other causalities not directly related to spasticity have been proposed such as musculoskeletal-derived pain, inhibition of the release of the neurotransmitters of pain mediate pain modulation, mediating an effect on central or peripheral sensitization instead of reducing spasticity by blocking nerve impulse relay [4,10,20,21]. AbobotulinumtoxinA/Dysport (BoNT) can be used to evaluate whether this interrelationship between spasticity and pain exists [4,20,21] because BoNT has a documented antispastic effect and has been utilized to modulate spasticity for 2 decades [22,23]. In the first part of the study, we examined the interrelationship between pain and spasticity by evaluating the effects of BoNT.

Despite the high prevalence of pain in children with CP, pain is unrecognized and as a consequence undertreated [4,5,6]. Difficulties in identifying pain might be because pain can originate from multiple tissues and organs, and CCP may have an impairment in the cognitive and physical ability to communicate. This might make it difficult to identify CCP due to limitations in the existing clinical diagnostic tools used to evaluate pain. [5]. Tools to identify pain and appropriate pain evaluation methods are needed in research and clinical practice. The methods used to evaluate pain should consist of multiple and validated “pain assessment tools to cover the various dimensions of pain” [5]. The first step in diagnostic clinical practice is a thorough description of symptoms and clinical findings. This might be the first step in making it possible for us to identify CCP in pain. Previous studies of the signs and symptoms of CCP in pain are sparse, and pain in CCP has not been adequately described [24,25,26].

The prevalence and intensity of the pain seem to increase with age and the severity of the disease in CCP [7,27]. The pain can, as mentioned, originate from multiple tissues and organs, but in most CCP, pain is located in the lower extremities [27]. Pain has been located in a few cross-sectional register studies with proxy reporting, but none of these by using the direct clinical examination. [7,27,28,29] The hip region is especially involved in the more severely affected and non-ambulatory children (Gross motor function classification system [GFMCS] 4-5) [7]. The hips contribute to high rates of pain and are the cause of difficulties in performing the activities of daily living such as positioning, sleeping, dressing, and undressing. [30]. Intraarticular bone surgery and extraarticular soft tissue procedures for hip subluxation are the (surgical) approaches to deal with hip pain because the pain has been perceived as secondary to spastic hip subluxation, and this has caused uncertainty in the origin of intra- or extra-articular causality of hip pain [13,20]. In two previous studies, extra-articular and intramuscular injection of BoNT has resulted in pain relief in spastic hip disease [9,13]. Hip luxation in CCP is traditionally perceived to be caused by the extra-articular spasticity of the muscles around the hip [31], thus injection of BoNT should alleviate intra-articular pain. However, reducing spasticity with BoNT does not seem to prevent hip luxation [32,33,34]. Injection of extra-articular BoNT to treat pain in the hip region will allow us to determine whether the pain is due to intra- or extra-articular factors. In the second part of the study, we set out to provide a detailed description of pain in CCP and how pain is manifested in CCP, including the location of pain in the lower extremity by direct clinical examination.

## 2. Results

### 2.1. Subjects and Treatment

**Figure 1:**
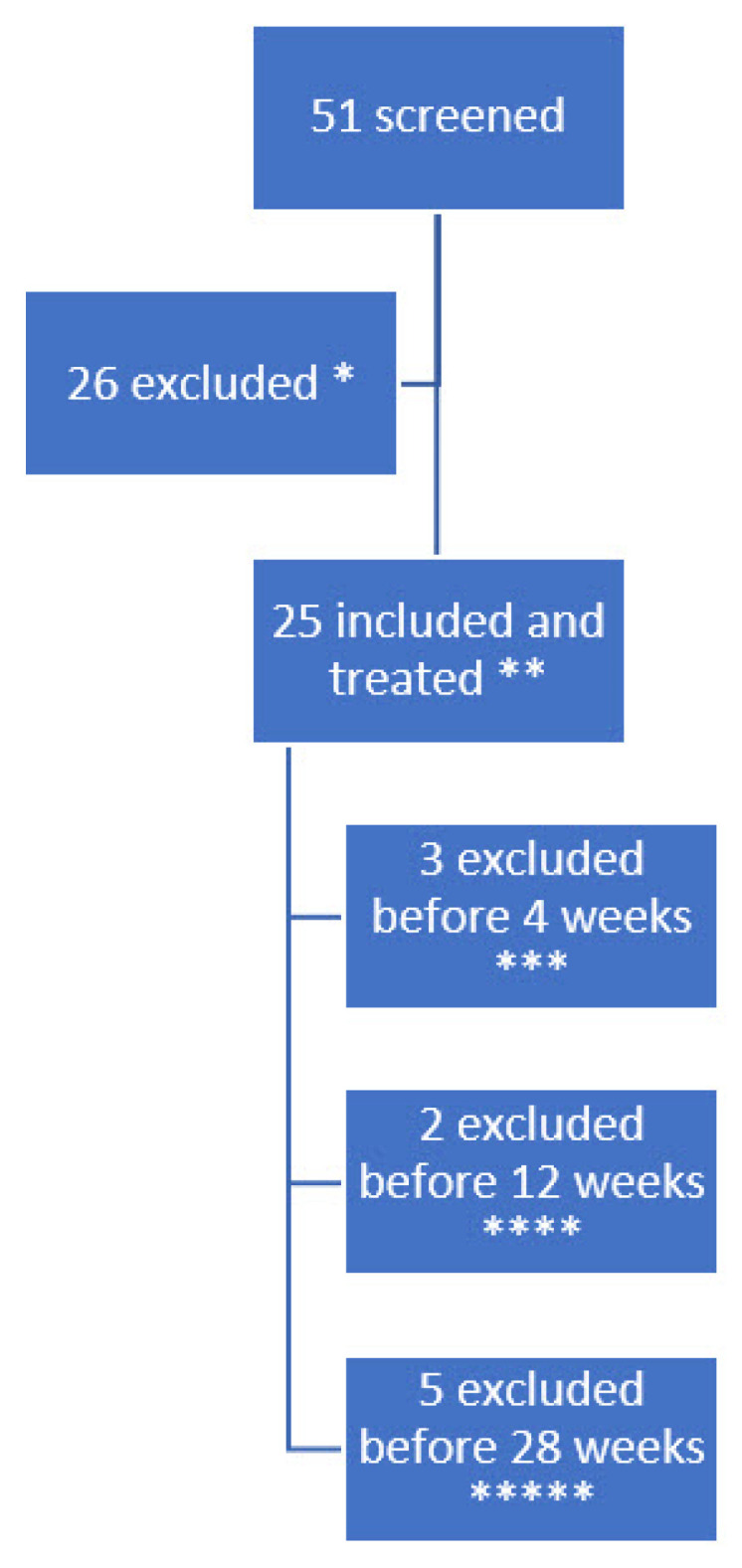
Timeline of the history of the subject’s participation. * A total of 20 subjects due to r-FLACC < 4, 2 due to other treatments, 4 did not want to participate, ** with r-FLACC > 4, *** two due to no effect and treated shortly after with another botulinum toxin injection treatment with higher dose and other muscles. One had a good effect but was treated with AboA which was not monitored adequately and was thus excluded. **** Two were excluded since pain reoccurred and had additional pain treatment and ***** five subjects were excluded due to deteriorating effect and were excluded to have another AboA injection.

Children with CP in our general service area were screened for eligibility. In a previous publication, we characterized the subjects including medical history and previous BoNT injections and described the inclusion process and history of withdrawals, the ultrasound-guided injections procedure including doses and targeted muscles, and the assessment methods for pain and functional changes using the goal attainment scale, quality of life, and adverse events [35]. Fifty-one of them had pain and were contacted through their caregivers for inclusion after a formal invitation by letter. After caregivers accepted participation, their medical records were screened according to the inclusion and exclusion criteria. If still eligible, the patients were examined for lower extremity muscular pain by the r-FLACC during pROM (≥4). Twenty patients had pain levels too low to participate, four patients declined to participate and two had other ongoing changes in medical treatment, hence were excluded. Twenty-five patients with spastic cerebral palsy and an r-FLACC pain level larger than or equal to four were included as subjects and received a single injection of BoNT. The majority had previous BoNT treatment (21/25). Twenty-two subjects were evaluated at a 4-week follow-up, 20 at 12 weeks, and 15 at 28 weeks. The total withdrawals were nine, whereas seven had a good but subsiding pain-relieving effect and there was no effect on pain in two subjects. One subject was treated with BoNT with good effect, but the storage temperature of the BoNT was not monitored adequately before injection and thus is excluded. Figure 1 shows a flow chart of the history of the subject’s participation and exclusion.

The average age was 9.1 years with a range from 2 to 17 (SD: 3.6) for the 22 subjects that were still included at the 4-week follow-up. The gender ratio (female:male) was 8:14. They were classified as unilateral:bilateral with a ratio of 3:19 (hemilegia: 3, diplegia: 9 and tetraplegia: 10). Table 1 shows a characterization of the subjects according to the GFMCS and other classifications.

**Figure 2:**
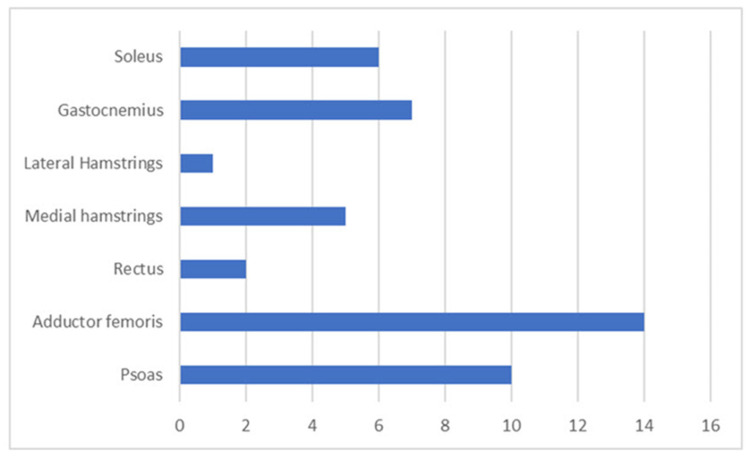
Distribution of the painful muscles of the lower extremity (X-axis: number of muscles, Y-axis: muscle name).

When evaluated by the r-FLACC for pain in pROM, the subject had located pain combination of one muscle (Adductor Femoris) for two subjects, two muscles groups (often Psoas of the hip, Adductor Femoris or the Gastronemius and Soleus muscle of the Gastrosoleus muscle of the calf) in seventeen, and three muscle groups (Psoas of the hip, Adductor Femoris combined with either Gastrosoleus of the calf or medial hamstrings or Rectus Femoris) in three. The location of pain related to muscles is shown in Figure 2.

Sixteen out of 22 (16/22) subjects received other pain-related medicine and other types of medical treatment. After an injection of AbobotulinumtoxinA/Dysport, medications were changed in 9/17, pain-related medications were changed in 9/17, and treatment for, i.e., epilepsy was changed in 6/17. Eighteen out of 22 had received previous botulinum toxin injections with an average of four injections. Fourteen out of 21 had had orthopaedic surgery. The specific levels of surgery and medical treatment are described in Table 2. All subjects received training; 21/22 were given training (physio and occupational therapy) 2.6 sessions per week on average.

### 2.2. Part 1: Relationship between Pain and Spasticity

We found a pain-relieving effect on localized muscle pain in 96% (21/22) of the subjects with a significant mean and maximum r-FLACC score reduction at 4 weeks, 12, and 28 weeks (*p* < 0.05) as described in detail in our previous study [36]. The subjects had a mean decrease in pain level evaluated by the r-FLACC scale of 2.68 (SD: 2.07), and a mean decrease in spasticity level evaluated by the MAS of 0.36 (SD: 1.97) when comparing the levels of pain and spasticity at baseline and after the first 4 weeks. When examining the relationship between the reduction of pain and the change in spasticity, we found no significant correlation using linear regression analysis (adjusted *R*-squared: 0.012, *p*: 0.28 for the MAS, adjusted *R-*squared: 0.012, *p*: 0.013, *p*: 0.68 for the TAR, and adjusted *R*-squared: 0.012, *p*: 0.013, *p*: 0.28 for the pROM). There was a decrease in daily pain level using the PPP of 0.95 (SD: 1.24) but no significant correlation to the decrease in spasticity (adjusted R^2^: 0.03, *p*: 0.45 for the MAS).

#### 2.2.1. Relationship between Pain and Effect of BoNT Dose

The subjects were treated with a mean dose of Abobotulinum toxin-A/Dysport of 17.3 units per kilo (SD: 6.7). When examining the relationship between the reduction of pain according to the r-FLACC after the first 4 weeks and the change in dose, we found no significant relationship using linear regression analysis (adjusted R^2^: −0.052, *p*: 0.92).

#### 2.2.2. Relationship between Spasticity and Effect of BoNT Dose

When examining the relationship between the reduction of spasticity after the first 4 weeks and the change in dose, we found no significant relationship using linear regression analysis (adjusted R^2^: −0.05, *p*: 0.35).

### 2.3. Part 2: Pain Location/Muscles and Regional Effect of BoNT

**Figure 3:**
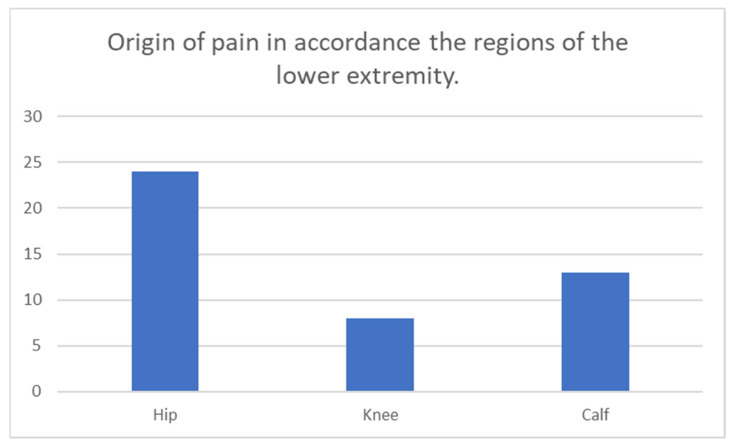
The origin of pain in the lower extremity (X-axis: name of the region, Y-axis: number of muscles in the region).

The treated lower extremity muscles categorized in regions are illustrated in Figure 3. Pain in the lower extremity was located in the hip, thigh, and calf regions, with a muscle distribution of 24/45, 8/45, and 13/45. We found a significant difference in effect in pain reduction after BoNT injection in pairwise comparison between-subjects thigh to the calf (*p*: 0.023) and hamstring and rectus (*p*: 0.023). However, after applying the Bonferroni correction, there was no significant difference (significance threshold for regions: *p*: 0.0125 and muscles: *p*: 0.008). Otherwise, there were no significant differences in the pairwise comparisons for effect on r-FLACC, PPP, or spasticity reduction even at a significance level of 0.05.

#### 2.3.1. Gender-Related Differences

We found no significant gender differences in baseline pain status, mod. Ashworth and Tardieu, range of motion, and BoNT doses and for the change in these parameters after 4 weeks.

#### 2.3.2. Behavioural Descriptors of Pain 

Caregivers described behavioural indicators of pain in their child when examined and evaluated by the r–FLACC. These behavioural indicators were related to specific changes in the child’s facial expression in 9/12, movements in the legs in 6/12, other specific activities in 6/12, verbal outbursts in 9/12, and consolability in 1/12. The behavioural indicators are given in Table 3.

When caregivers evaluated their child for daily pain by the PPP, they noted almost all the subjects were tensing up/stiffening/having spasms when in very severe pain and severe daily pain. This was predominantly most of the time or always in more than half of the subjects. When in very severe pain, the children also frowned their eyebrows/looked worried, and they were difficult to console, had sleep disturbances, grimaced, looked frightened, or were squirming in more than half of the subjects. For all levels of pain, pain can be recognized by the child tensing up/stiffening/having spasms, grimacing, frowning eyebrows, having sleep difficulties, or not being as social. The full answers to the questionnaire can be seen in Supplement Information.

#### 2.3.3. Parental Interviews/Specific Questions

There were 5/9 subjects evaluated using the WBS and 4/9 using the NRS. Caregivers evaluated their child’s pain level as low in 3/7 and high in 4/7. Caregivers evaluated that their child was able to distinguish pain from other sensations in general in 6/7 and daily living in 7/8. When inquired about the mild and worst pain in a week, this was low in 3/6 and high for 3/6 for mild pain during an average week, and worst pain during an average week was low in 1/6 and high for 5/6. The specific questions in the interview can be seen in Supplement Information.

#### 2.3.4. Parental Evaluated Patient-Specific Relevant Initial Goals Discovered during the Interviews

Caregivers described 92 relevant, initial goals, thereby describing the child’s and/or the family’s relevant problems related to pain. Thirty-two problems (32/92) were related to sleep. Out of these, 7 (7/32) were related to falling asleep, 18 (17/32) pertained to awakenings during the night, 3 (3/32) were related to sleep length, and 5 (5/32) were concerned with sleep quality. Three (3/92) problems were related to “better function” with a focus on the range of motion of ankle dorsiflexion. Six (6/92) problems were related to “falling in daily living” with a focus on the number of falls a day. Fifteen problems (15/92) were related to mobility. Out of these, five (5/15) were related to standing function, six were on using walking frames (3/15) and cycling (3/15), two were related to independent walking (2/15), and two to general activity (2/15). Twenty-one problems (21/92) were related specifically to pain. Three (3/21) were related to pain level, nine (9/21) were on the number of episodes of pain, and nine to pain in a specific activity (9/21). Fifteen problems (15/92) were related to “others”. Three (3/15) were related to using orthotics, three were to put on shoes (3/15), three related to the number of epileptic seizures (3/15), and six to general well-being (6/15). The specific problems are described in Table S1. When asked whether the pain was the cause of impediments in daily activities, caregivers answered that the pain did not prevent the performance of activities for 3/22, in a few activities for 3/22, in some activities for 8/22, and in most activities for 8/22.

## 3. Discussion

In this study, we evaluated a relationship between changes in localized pain and spasticity y level. In part 1 of the study, we found a significant and clinically meaningful pain-relieving effect after one injection treatment of BoNT which was highest at 4 weeks and significantly maintained at 28 weeks. Shearer et al. (2023) found that the course of pain intensity in children with CP receiving usual care is stable in both the short and long term. Moreover, when receiving BoNT, significantly lower pain intensities are reported after one month [26,37]. This corresponds with our findings. Secondly, for the relationship between changes in localized pain and spasticity level, we found no significant correlation between the two after BoNT-mediated pain reduction. We expected that changes in pain and spasticity levels were correlated [4,10,20] because it is generally assumed that pain and spasticity are closely interrelated [20,27] (Table 1). Our findings suggest that pain reduction, and thus pain, is mediated by mechanisms other than inherent spasticity. A previous study by Flannigan et al. (2020) found an interrelation between overall pain and spasticity levels but was unable to find (a weak) correlation between localized pain and spasticity levels [18]. The authors concluded that a more subjective measurement of hypertonia was associated with greater pain levels [18]. This supports the notion that the pain might not be caused by spasticity but is associated with overall hypertonia and severe CCP [19,20]. In our study, we were unable to find a correlation between the dose of BoNT and pain reduction, which adds indirect support to this. A focus on spasticity in pain management treatment in CCP using treatment with BoNT might explain why previous studies have been unable to identify a relationship between pain and spasticity [19,20]. If pain cannot be relieved by reducing spasticity via blocking nerve impulse relay, then pain in CCP might be caused by simple muscular overload or inhibition of the release of the neurotransmitters of pain [4,10,20]. This finding is significant because the primary intervention for musculoskeletal pain entails non-surgical treatments such as therapeutic exercise, weight loss, and patient education as first-line interventions [10]. In our first study, we found a clinically relevant and significant pain-relieving effect of BoNT [35], thus indicating that the use of BoNT could be expanded to include a specific role in pain management, but the evidence for utilizing BoNT in musculoskeletal pain is contradictory [10]. This warrants further studies with a stronger design because the pain in CCP is undertreated [24] and is even more disabling than the movement disorder itself [8,38]. Self-reported pain in CCP is the primary determinator for diminished quality of life [4,5], [38,39,40]. In part 1 of this study, we were unable to detect an expected BoNT dose-dependent decrease in spasticity [41,42,43,44,45]. At first glance, this is unexpected because we usually assume a relationship between the dosing of BoNT and modulation/reduction of spasticity. However, this might also be due to our delivery of BoNT. The majority of injections were given in the psoas component of the iliopsoas muscle at the level of the hip joint in the tendinous/myotendinous junction. Thus, the BoNT was not delivered directly into the muscles and was, therefore, unable to facilitate spasticity reduction. However, this could also be due to the well-known limitations in reliability and content validity using the evaluation methods for measuring spasticity [36,46,47]. We compensated for the limitations by using only one specially trained rater for all evaluations of spasticity and pain to improve the reliability of our measurements. In this study, we assumed a relationship between reduction of pain and spasticity to interpret a relationship between pain and spasticity, and this has not been established. In conclusion, we were unable to demonstrate a relationship between localized pain and spasticity. However, our findings should be interpreted cautiously with the above limitations in mind.

The motivation to perform part 2 of this study was that pain in children with CP is often undetected by healthcare professionals and, as a consequence, undertreated [4,5,6]. The reason is that pain can originate from multiple tissues and organs in CCP with potentially diminished communication skills due to impairments in cognitive and physical ability. In this study, five centres and multiple doctors screened and identified potential candidates for CCP in pain. However, during the first two years of the study, we were able to include only a few subjects. After giving detailed oral and written information as outlined in this article to a selected group of doctors, they were able to identify several subjects in the course of a few months. We suggest our primary difficulties in recruiting patients might also be due to limitations in the existing framework for clinical diagnostic pain evaluation in CCP [5], and for this reason we provide in this article a detailed description of the tell-tale signs and symptoms of pain provided by the caregivers. This was described as a child’s pain-related problems in the parental interviews. This information was gained through our systematic pain questions and the clinical evaluation by a single rater, which enabled us to obtain descriptions of how pain manifested itself in daily living, psychometrics, and medical history. We found that (chronic) pain is not only associated with decreased physical functioning such as tripping/falling but also with disturbances in sleep, increased fatigue, and impediments in the child’s school and social life [35]. Previous studies of the signs and symptoms of CCP in pain are sparse in number and the pain is described incompletely [24,25,26]. In general, appropriate pain evaluation methods should entail multiple and validated pain assessment methods to cover the various dimensions of pain [5]. Especially when examining pain in communicative and non-communicative children with CP, thorough and adequate pain evaluation is challenging since they, besides having a physical disability, also are challenged with cognitive, perceptive, and communicative impairments. This warrants appropriate, accurate, and standardized pain evaluation methods to meet these challenges—preferably by self-reporting and using multiple and validated ‘pain assessment tools’ [5,37,48]. We found that the *pediatric pain profile* covers the described symptoms and signs of daily pain fairly adequately. This corresponds with Caraveu et al. (2022), who found the pediatric pain profile (and r-FLACC) acceptable for the evaluation of pain in children with CP [49]. Only one previous study has provided partial descriptions of CCP in pain which included symptoms and signs, such as frequent nightly awakenings, irritability with feeding, facial grimacing, and crying with movement such as with diaper changes [24]. We hope this article will provide a case-based guide for identifying CCP in pain. Importantly, we found some counter-intuitive signs of pain such as laughing. This tell-tale sign was provided by the caregivers, and in our experience, it is important to ask the caregivers about specific tell-tale signs of pain when CCP are examined; however, previous studies have indicated that proxy-reported pain tends to overestimate pain in comparison to self-reporting [50]. In this study, almost all evaluations were performed by the caregiver, but when given the choice, 2/6 teenage subjects self-reported on the quality of life questionnaire [35]. We also noted that many of the medications taken by CCP were directly pain-relieving or relieved symptoms of pain such as medicine for constipation, sleeping pills, and muscle relaxants. Caregivers primarily treated their child with non-pharmacological and non-surgical pain management such as massage (7/17) [51]. This might also constitute a tell-tale sign of pain. We were unable to detect significant gender-related differences in our outcome parameters, which is contradictory to previous studies, which have demonstrated higher pain prevalence and intensity in females [25,52].

**Figure 4:**
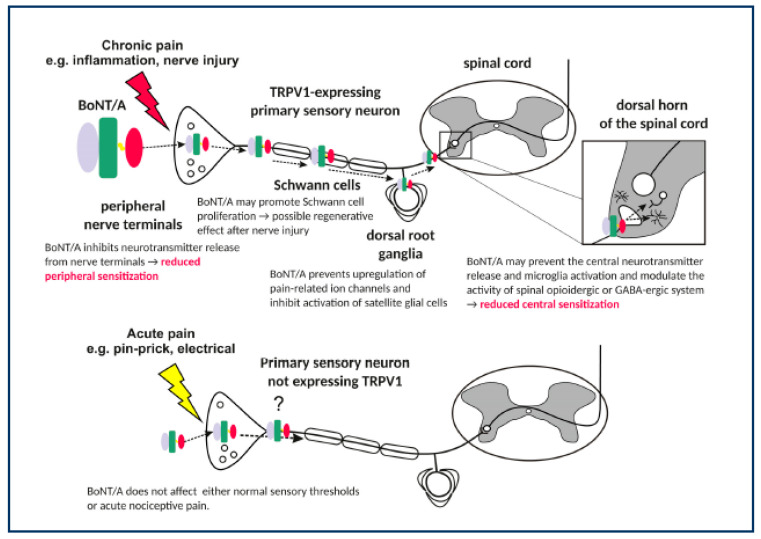
Actions of BoNT/A along the pain pathway. (Courtesy of Matak I, Bölcskei K, Bach-Rojecky L, Helyes Z. Mechanisms of Botulinum Toxin Type A Action on Pain. Toxins (Basel) [24].

In part 2 of this study, the pain was situated in the hip region in over half of our subjects. The subjects were almost equally distributed in the GMFCS levels 1–2 and 4–5 (8/12) and a majority of our subjects had bilateral CP (19 out of 22). This is in contrast to other studies, where the pain was located distally in the lower extremity in the less affected CCP [7,27]. We ascribe this to our main focus on pain in the evaluation using several methods to capture dimensions and localization of the pain in every joint and muscle group [5,35]. We found a significant and clinically meaningful pain-relieving effect with almost full recovery after one ultrasound-guided injection treatment of Abobotulinum toxin-A/Dysport. Hip pain in clinical orthopaedic practice is often perceived as a bony intra-articular problem and treated surgically as such when found indicated, thus our finding could have implications for future treatment. We also speculate whether our positive effect is due to our focused and localized evaluation because previous studies have demonstrated a moderate level of evidence for pain (OCEBM/Oxford Centre for Evidence-Based Medicine level II) [10,19,20,35,39]. Presuming our single intervention mediated the clinically relevant and almost full pain-reducing effect, this would mean that this type of hip pain constituted extra-articular, thus soft tissue-derived pain. All of our subjects were without clinical or radiological signs of intra-articular hip pathology. Moreover, there was no significant decrease in spasticity in our subjects. The latter finding is supported by Lundy et al. (2009), who found a highly significant effect of extra-articular BoNT in non-ambulatory CCP [13]. They ascribed the effect to either reduction in muscle tone, the direct effect of reducing mechanical stimulus to the pain afferent system in the soft tissues surrounding the hip joint, directly reducing the increased compression on the blood vessels and nerves and causing a reduction in the nociceptive stimulus, or direct peripheral analgesic and anti-inflammatory activity [13]. If there is no relationship between pain and spasticity, then pain in CCP might be due to either simple muscular overload or inhibition of the release of the neurotransmitters of pain. Other mechanisms of pain have been suggested by experimental and clinical studies. Matak et al. (2019) suggested actions of Botulinum toxins along the pain pathway as suggested chronic pain-reducing mechanisms for pain mediated by, i.e., inflammation or nerve injury [21]. BoNT acts at the terminal nerve endings by inhibiting neurotransmitter release from the nerve terminals, thus could reduce peripheral sensitization, and on the Schwann cells in the afferent nerves by promoting Schwann cells proliferation with a potential regenerative effect after nerve injury, on the dorsal root ganglia by preventing upregulation of pain-related ion channels and inhibiting activation of satellite glial cells, and in the dorsal horn of the spinal cord by preventing the central neurotransmitter release and microglia activation and modulate the activity of spinal opioidergic or GABA-ergic system, thus could reduce central sensitization [21]. Our findings would suggest that the action at the terminal nerve endings by inhibiting neurotransmitter release from the nerve terminals is not mediated by the spasticity reduction, but by the anti-inflammatory effect. In this study, BoNT could have acted peripheral and/or central pain-modulating mechanisms, but seem not to be mediated reduction of spasticity by blocking nerve impulse relay [20]. Figure 4 illustrates the potential actions of BoNT along the pain pathway, which BoNT could affect.

The symptoms of pain in CCP resemble musculoskeletal pain, a broad array of symptoms including body aches, malaise, stiffness, fatigue, and sleep disorders, and would suggest musculoskeletal overload as causal of the peripheral mediated pain indicated with a red arrow in Figure 4 [7,8,38,53]. Further studies examining different mechanisms of pain relief and the pathophysiology of pain will help identify the mediating mechanisms and have a direct effect on treatment [20].

## 4. Conclusions

In conclusion, we were unable to demonstrate a relationship between pain and reduction of spasticity. Pain in CCP can be discrete and overlooked, but consulting caregivers for tell-tale signs of pain, localized physical examination to locate the specific origin, and evaluating prescribed medicine aids in detecting the signs of pain. The pain was overrepresented in especially bilateral CCP but equally distributed in both GMFCS 1-2 and 4-5. In the hip region, pain seems to have a soft tissue/extra-articular origin.

## 5. Materials and Methods

### 5.1. Population

Pediatric patients with predominantly spastic CP were recruited as a convenience sample. Inclusion criteria were CCP from all GMFCS levels and between 2 and 18 years of age. They were either botulinum toxin naïve or had a latency period of 6 months from the last injection and had at least moderate pain when evaluated by the revised face, legs activity, cry, consolability scale (r-FLACC ≥ 4) during passive range of motion (pROM). The pain response determined the candidate muscles for injection with BoNT. Subjects were excluded if they had fixed contracture or severe athetoid/dystonic afflictions, or if modifications in their ongoing treatment that would affect the pain status had either taken place 3 months before the BoNT injection or during the evaluation period (antispastic or pain medication given orally or by injection or if subjects had interfering surgical procedures performed).

### 5.2. BoNT Injections

One single ultrasound-guided intramuscular injection of BoNT was administered at the discretion of the treating physician. BoNT dosages were determined by the treating physician, who injected 30 units per kilo (U/kg) in CCP with bilateral CP and 15 U/kg with unilateral CP, with a maximum dose of 1000 units. Small and large muscles were injected within a range of 3–6 U/kg and 8–12 U/kg, respectively. Small muscles were defined as muscles with an ultrasound-measured muscle thickness of less than 0.95 cm at the injection site and large muscles as muscles larger than 0.95 cm at the injection site [54,55]. Five hundred units of Abobotulinum toxin A/Dysport were diluted in 2.5 mL sterile NaCl in sterile syringes. Targeted muscles were determined after prior evaluation of pain response measured by r-FLACC during pROM.

### 5.3. Clinical Assessments

The subjects were assessed by observational pain evaluation and clinical examination for spasticity in the first part of the study. In the second part, subjects and caregivers answered a questionnaire for daily pain at baseline before injection and after 4 weeks. The interview consisted of a series of oral and written questions focusing on the characteristics of pain.

### 5.4. Part 1

#### 5.4.1. Assessments of Spasticity and Passive Range of Motion

One single therapist performed all the examinations. Evaluation of the lower extremities was tested systematically in the supine position by clinical examination of passive range of motion (pROM), Modified Ashworth Scale (MAS), and Modified Tardieu Scale (MTS) of the treated muscles. For the modified Tardieu, we evaluated the angle of the very slow (V1) and a quick stretch (V3) [36]. We measured pROM, MAS, and MTS in a standardized manner following recommendations and using a two-arm goniometer when appropriate [46,56,57].

#### 5.4.2. Assessments of Pain

The observational pain tools were utilized to determine the presence of localized pain by the r-FLACC and the pediatric pain profile (PPP) for daily pain.

#### 5.4.3. Localized Muscular Pain Evaluated by the Revised Face, Legs Activity, Cry, Consolability Scale (r-FLACC)

Initial clinical evaluation entailed pROM of all muscles of the lower extremity to identify potential localized muscular pain using the r-FLACC. The r-FLACC scale is a validated observational and behavioural pain intensity tool with five measurement categories with a 3-point ordinal scale (0–2), ranging from 0 to 10 possible points. Each category entails a description of behavioural signs in the facial expression, legs, activity, cry, and consolability [58]. The r-FLACC scores were evaluated during the examination and were videotaped systematically using two iPads, thus enabling us to re-evaluate the subject in the frontal and sagittal view [59]. The caregivers added a unique descriptive ‘pain’ behaviour of the child, i.e., verbal outbursts, tremors, increased spasticity, jerking movements, and respiratory pattern changes to ensure that our ratings were individual and accurate. The caregivers added unique behavioural pain descriptors of the child. This was added to improve reliability and ensure that our ratings were individual and accurate [58]. The localized pain was evaluated for the treated muscles at baseline and after 4 weeks. Since injections of BoNT are presumed to have a localized effect, our primary endpoint was the change in the r-FLACC score. This was evaluated during the passive range of motion of the targeted muscles and when BoNT would peak in effect for spasticity reduction, namely at four weeks [22].

#### 5.4.4. Daily Pain Is Evaluated by the Pediatric Pain Profile (PPP)

The PPP is a validated 20-item behaviour rating scaled questionnaire for assessing pain behaviour and monitoring responses to treatment in children with neurological impairments [60,61,62]. Each item has a 4-point ordinal scale (0–3) with a total score ranging from 0–60. The PPP is a caregiver-held tool to evaluate everyday pain.

### 5.5. Part 2

#### 5.5.1. Pain Interviews and Specific Tell-Tell Signs of Pain

We interviewed subjects and caregivers by asking them a series of oral and written questions. We inquired specifically about pain when the child was examined, the child’s pain perception, and the level of pain in daily life. The caregivers answered the questions either with dichotomous answers (yes/no) or by using one of two numeric rating scales, the Wong-Baker Faces Pain Rating Scale (WBS) grading from 1–6 and the Numeric Rating Scale (NRS) grading from 1–10. The choice of the scale was determined according to either preference or age. The WBS was chosen for subjects less than 8 years of age and the NRS for subjects greater than 8 years. We categorized information on the two scales into three levels: no pain (WB: 0, NRS: 0), low pain (WBS < 5, NRS < 7), and high pain (WBS > 4, NRS > 6). The choice of self-reporting of pain was determined by age due to the validity of the NRS suitable for assessing pain intensity in youths with physical disabilities between the ages of 8 and 20 years, whereas the Wong–Baker Faces Pain Rating Scale is better for the younger subjects [61,62,63]. The oral and written questions in the interview are listed in Supplement Information. During the interview, we also inquired about the individual pain indicators that the caregivers perceived as signs of pain and subsequently utilized them in the r-FLACC evaluation. The caregiver was also asked to evaluate if and how the child had impediments and which activities in everyday living were compromised and needed improvement. The latter information was retrieved as the initial goals or functional changes when evaluated by the goal attainment scale (GAS) using the specific, measurable, achievable, relevant, and timed (SMART) principles. During the initial interview, two to three pain-related, unweighted, individual, and relevant GAS goals were defined in detail using the SMART principles set for each child [64].

#### 5.5.2. Statistical Analysis

Linear regression analyses were performed with pain as the dependent variable and spasticity using the mod. Ashworth and Tardieu, range of motion, and BoNT doses as independent variables. We also tested spasticity as the dependent variable and BoNT doses as independent variables. To test for potential relationships, the *p*-values of each variable and the adjusted *R*-squared values were evaluated. *p*-values of ≤0.05 were considered statistically significant. Assessment for the variance of the residuals/heteroscedasticity was evaluated by the histograms of the residuals and normal probability plots. For evaluating gender differences, we performed an independent sample t-test for baseline pain status, mod. Ashworth and Tardieu, range of motion, and BoNT doses and for the change in these parameters after 4 weeks. Assessment for the difference in variance was evaluated by Levene’s test. Bonferroni corrections were applied when multiple tests were performed to reduce type I error. In a previous study, the sample size was estimated to be 16 subjects for evaluation of the effect of the injection treatment on pain level evaluated by modified r-FLACC [35]. All other tests were performed using IBM SPSS Statistics, Version 25 (IBM, Richmond, VA, USA). The Regional Committee on Health Research Ethics and the national medical agency approved the study (H–17041772 and EudraCT number 2017-004497-33). We obtained oral and written consent from the caregivers, and the study was conducted according to national guidelines and the Helsinki Declaration. The study was investigator-driven and supported by Ipsen AB.

## Figures and Tables

**Table 1:** Characterization of the subjects according to the GFMCS and other classifications of the subjects that were still included at the four-week follow-up (number of subjects in category).

GFMCS		Gender		Affected Side		Affected Body Part	
1	4	Male	14	Left	7	Hemiplegia	3
2	4	Female	8	Right	15	Diplegia	9
3	1					Tetraplegia	10
4	5						
5	8						

**Table 2:** Description of types of oral medication, previous invasive procedures including regions where these were performed, and other types of interventions by therapists (number of subjects in category/* regions of the lower extremity).

Types of medication	Muscle relaxants	Sleeping pills	Epilepsy medicine	Medicine for constipation
	7/15	3/16	9/16	7/16
Medicine for pain	Pain-relieving medicine	Paracetamol		
	6/16	9/16		
Invasive procedures	Previous botulinum toxin injections	Number of injections	Previous surgery	
	18/22	4	14/21	
Regions *	Hip region	Thigh region	Knee region	Calf region
	8/21	1/21	7/21	7/21
Other interventions	Other treatment/physical training	Physical/occupational training	Formalized physical training/occupational training per week	
	22/22	21/22	2,6	

**Table 3:** Specific and behavioural tell-tale signs of pain are reflected in facial expression, movement of the legs, general activity, crying, and inconsolability.

Subject Number	Face	Legs	Activity	Crying	Consolability
Subject 1	Whinces his eyes	Pulls left leg up towards the trunk	Hits himself in the head	Making sounds and grunts	
			Holds his hands on his ears		
Subject 2	Wrinkles eyebrows		Curls into a ball	Says ‘Aw’	
			Resists examination		
Subject 3	Grimacing	All muscles tense up	Tries to move away	Starts crying/screaming	
Subject 4	Withdraws/become quiet	Legs tense up			
Subject 5			Starts squirming	Initial crying subsides	
Subject 6	Starts smiling			Starts laughing	
				Says ‘Aw’	
Subject 7			Starts squirming		Seeks specific caregiver
Subject 8	Starts smiling			Starts laughing	
Subject 9	Wrinkles eyebrows	Legs tense up		Stops making sounds	
	Sticking tongue in and out				
Subject 10	Starts smiling			Starts laughing	
				Says ‘Aw’	
Subject 11	Starts smiling	All muscles tense up		Starts laughing	
				Says ‘Aw’	
Subject 12		All muscles tense up	Raises hands		
			Starts kicking		
			Resists examination		

## Data Availability

For further interest, inquiries for data can be sent to the corresponding author.

## Supplementary Materials

The following supporting information can be downloaded at: https://www.mdpi.com/article/10.3390/toxins15020152/s1, Supplement Information: The specific oral and written questions in the interview; Table S1: Notes from the interview with the caregivers and subjects pertaining to pain.
