# Supplementary material for: The Relationship between Pain and Spasticity and Tell-Tale Signs of Pain in Children with Cerebral Palsy
Source: Toxins (Basel). 2023 Feb 13;15(2):152. doi: 10.3390/toxins15020152 (PMC9967793; doi:10.3390/toxins15020152)
Supplement: Supplementary file 1 [file toxins-15-00152-s001.zip › toxins-2098190-supplementary.pdf]

toxins

Article

The Relationship Between Pain and Spasticity and Tell-Tale Signs of Pain in Children with Cerebral Palsy

Christian Wong

Supplement Information.

The specific oral and written questions in the interview:

What was your child's pain level when examined?

Is your child able to distinguish pain from other sensations in daily life?

Is your child able to distinguish pain from other sensations in general?

What is the level of mild pain in an average week?

How much does the pain affect your child's daily living?

Describe the pain your child is experiencing? (preferably in as much detail as possible)

How long has your child been experiencing this pain?

When does this pain usually occur?

How often does this pain occur, e.g., all the time, every day, weekly, etc.?

What can usually help?

Table S1. Notes from the interview with the caregivers and subjects pertaining to pain.

	Describe the pain your child is experiencing (preferably in as much detail as possible)	How long has your child been experiencing this pain?	When does this pain usually occur?	About how often does this pain occur, e.g. all the time, every day, weekly, etc.?	What can usually help?
Subject 1	Crying - when is mounted in a standing frame	Keeps the left leg bent 1 year: does not stretch the leg out.<br>Last 5 months have seemed quiet and less happy.	In the standing frame<br>When stretching knee		
Subject 2		Within the last year. Has been increasing with age	When in a Walker (functional walking aid)<br>When muscle stretching	Every Day	Massage
Subject 3		After surgery 3 years ago	Activities - mobilization<br>Undressing/dressing	All the time, every day	Position the subject<br>Using a chain duvet
Subject 4	Hyperesthesia of the left big toe	The last few years - especially after hamstring surgery<br>since subject has become more active	After active day<br>When wearing socks or orthosis	Very often	Massage v. footsole/midfoot
Subject 5	Pain in the right groin.<br>Experiencing legs collapsing due to pain, which leads to falls	After surgery 3 years ago	Both by palpation<br>and in movement in ROM	Every Day	Oral pain medicine with paracetamol
Subject 6	Lower leg/calf - tenses up	Always	No triggering factors	Several times a day	Oral pain medicine with paracetamol<br>Soothing the child/Hot baths<br>Stretching the leg
Subject 7	Pain in the back and hip	Always<br>Especially after no walking support 2 years ago	When he is going to sleep<br>When relaxing	Every Day	
Subject 8	Pain in legs in the hip, hamstring, and ankle region	Always	When standing<br>In activity	When active	Massage
Subject 9	Leg pain<br>Unilateral	Always	When activating right calf muscles<br>When wearing shoes	Every Day	Avoid activities activating right calf

Subject 10	Pain in hip and calf<br>Unilateral	Dependant on level of activity	Occur at night when going to sleep<br>When running and playing foot-ball	Every second day	
Subject 11	Pain in the leg<br>Unilateral	Last three years	In activity/physical straining	Every Day	Heat<br>Decrease physical activity
Subject 12	Pain in shoulder and calf	Always	In activity/physical straining	2-3 times/week	Injections and Botulinum toxins<br>Massage and foot creme
Subject 13	Pain in calfs	Always<br>Especially when growth spurt started	After activity/physical straining	Every Day	Massage and muscle stretching<br>Rest for 2 days after strenuous ac-tivity
Subject 14	Groin pain	Caregivers noticed when subject was a toddler			Massage and heat
Subject 15	Stabbing pain in knee and adductors of the hip<br>Unilateral	During the last 4 months	Not activity related	Always	Lying with pillow under leg
Subject 16	Both legs - accentuated in adductors and heel<br>Unilateral	During the last 6 months	Activity related	Always	Foot massage and muscle stretch-ing<br>Lying with pillow under leg
Subject 17	Hip pain<br>Unilateral	Always	Post-activity related	1-2 times/week	Caregivers soothes child
Subject 18	Hip pain<br>Bilateral	For many years - accentuated the last 1-2 years	While sitting in chair	1-2 times/week	Massage and foot creme<br>repositioning in chair
Subject 19	Hip pain<br>Unilateral	For a year	Changing position from lying to sitting	Every Day	Heat and distraction<br>Oral pain medicine with paraceta-mol
